# A *CCL5* Haplotype Is Associated with Low Seropositivity Rate of HCV Infection in People Who Inject Drugs

**DOI:** 10.1371/journal.pone.0156850

**Published:** 2016-06-15

**Authors:** Kristi Huik, Radko Avi, Merit Pauskar, Eveli Kallas, Ene-Ly Jõgeda, Tõnis Karki, Kristi Rüütel, Ave Talu, Katri Abel-Ollo, Anneli Uusküla, Andrew Carrillo, Sunil K. Ahuja, Weijing He, Irja Lutsar

**Affiliations:** 1 Department of Microbiology, Institute of Biomedicine and Translational Medicine, University of Tartu, Tartu 50411, Estonia; 2 National Institute for Health Development, Tallinn 11619, Estonia; 3 Institute of Family Medicine and Public Health, University of Tartu, Tartu 50411, Estonia; 4 Department of Medicine, University of Texas Health Science Center at San Antonio, San Antonio, Texas 78229–3900, United States of America; 5 Veterans Administration Center for Personalized Medicine, South Texas Veterans Health Care System, San Antonio, Texas 78229, United States of America; University of Malaya, MALAYSIA

## Abstract

**Objective:**

The role of CC chemokine receptor 5 (CCR5) and its ligand CCL5 on the pathogenesis of HIV infection has been well studied but not for HCV infection. Here, we investigated whether *CCL5* haplotypes influence HIV and HCV seropositivity among 373 Caucasian people who inject drugs (PWID) from Estonia.

**Methods:**

Study included 373 PWID; 56% were HIV seropositive, 44% HCV seropositive and 47% co-infected. Four *CCL5* haplotypes (A-D) were derived from three *CCL5* polymorphisms (rs2107538/rs2280788/rs2280789) typed by Taqman allelic discrimination assays. The data of *CCR5* haplotypes were used from our previous study. The association between *CCL5* haplotypes with HIV and/or HCV seropositivity was determined using logistic regression analysis.

**Results:**

Possessing *CCL5* haplotype D (defined by rs2107538A/rs2280788G/rs2280789C) decreased the odds of HCV seropositivity compared to those not possessing it (OR = 0.19; 95% CI 0.09–0.40), which remained significant after adjustment to co-variates (OR = 0.08; 95% CI 0.02–0.29). An association of this haplotype with HIV seropositivity was not found. In step-wise logistic regression with backward elimination *CCL5* haplotype D and *CCR5* HHG*1 had reduced odds for HCV seropositivity (OR = 0.28 95% CI 0.09–0.92; OR = 0.23 95% CI 0.08–0.68, respectively) compared to those who did not possess these haplotypes, respectively.

**Conclusions:**

Our results suggest that among PWID *CCL5* haplotype D and *CCR5* HHG*1 independently protects against HCV. Our findings highlight the importance of CCL5 genetic variability and CCL5-CCR5 axis on the susceptibility to HCV.

## Introduction

A major role of chemokines is to promote leukocyte migration in order to participate in immune surveillance and innate/adaptive immune responses. Cysteine-cysteine chemokine ligand 5 (CCL5; formerly known as RANTES–regulated upon activation, normal T cell expressed and secreted) is a CC chemokine that binds to a variety of chemokine receptors including the major HIV co-receptor CCR5 [1]. On one hand, it poses antiviral activity by suppressing the entry of R5-tropic HIV strains by down-regulating CCR5 receptor expression through its occupancy of T cells and macrophages [2, 3]. On the other hand, CCL5 exhibits viral replication inhibition in vitro [4].

CCL5 also plays a critical role in the recruitment of T cells into the liver and, through that, influences the progression of chronic hepatitis C infection [5]. Chemokines such as CCL3 and CCL4 can contribute to the provision of CD4 T cells to help optimal CD8 T cell priming [6]. The participation of CCL3, CCL4 and CCL5 in the early T cell response to infections including HIV and hepatitis C virus (HCV) is crucial [7–9].

The polymorphisms in the promoter and first intron region of *CCL5* affect the expression of CCL5 and influence susceptibility as well as disease progression of HIV infection [10, 11]. The single nucleotide polymorphisms (SNPs) of *CCL5* and different haplotypes (depending on studied SNPs) have been associated with susceptibility to HIV and with the disease progression in HCV infection with various results (**S1 Table**). Most of these associations between *CCL5* variability and acquisition have been found in the context of sexual or vertical transmission. We have previously evaluated the associations between the receptor of CCL5 (CCR5) and another ligand for CCR5 (CCL3L1) genetic variability and the susceptibility to HIV and HCV among PWID in terms of co-infection. We showed that a higher *CCL3L1* copy number than population median decreases the odds of HIV positivity while *CCR5* human haplotype (HH) G*1 decreases the odds of HCV seropositivity [12, 13].

In Estonia a rapid increase in the prevalence of hepatitis B (HBV) and HCV infection among people who inject drugs (PWID) was seen by the end of 1990s and was followed by the HIV epidemic in August 2000 (www.terviseamet.ee, [12]). As of now, approximately half of PWID are infected with HIV and more than 70% with HCV and HBV, providing an opportunity to look into the association between host factors and the abovementioned infections in the context of co-infections. Considering the potential role of CCL5 on HIV and HCV infection, here, we aimed to investigate associations between *CCL5* haplotypes and susceptibility to HIV and/or HCV infection in Caucasian PWID.

## Material and Methods

### Subjects and laboratory analyses

A total of 373 Caucasian PWID (300 male; 55 female and 18 gender unknown; median age of 26 years) were recruited in 2006 and 2007 from two syringe-exchange programs (n = 270) using a respondent-driven sampling [14, 15], and from three Estonian prisons (n = 103). The duration of intravenous drug use (IVDU) was available for 67% subjects. All study subjects were Caucasians from Estonia and seven subjects (3.4%) reported that they had received or were receiving antiretroviral therapy. The data of HCV treatment was not available. Sample collection and the detection of HIV, HCV and HBV was performed as described previously [12].

The demographic characteristics (HCV serostatus, median age and gender) of subjects were similar both from the syringe-exchange programs and the prisons (p>0.1). It was not feasible to recruit HIV negative subjects from prisons. Prisoners were determined as PWID by their indication of previous drug use because, according to official sources, there is no drug use inside the prisons. The data on the duration of IVDU was not available for persons recruited from prisons but was recorded for the majority of PWID (92%) from the syringe-exchange programs [12].

Genomic DNA was extracted from whole blood using the Qiagen QIAamp DNA minikit (Qiagen, Hilden, Germany). CCL5 polymorphisms G-471A (rs2107538), G-96C (rs2280788) and TIn1.1C (rs2280789) were detected by the TaqMan Allelic Discrimination assay (Applied BioSystem, Carlsbad, CA, USA). The data on *CCR5* haplotypes was available from our previous study [12].

### Statistical analyses

The program R 2.8.1 [16] was used and a p value of <0.05 was considered statistically significant. Differences in the distribution of *CCL5* haplotypes between study groups were compared by the Fisher exact tests as appropriate. Univariate and multivariate logistic regression models (including step-wise logistic regression analysis with backward elimination) were constructed to determine the associations of *CCL5* haplotypes with HIV and HCV serostatus. Age, gender, HBV serostatus and the duration of IVDU were included as covariates. The study was approved by the Ethics Committees of Tallinn, University of Tartu and University of Texas Health Science Center at San Antonio; all participants signed the informed consent following the Helsinki declaration.

## Results

### Study population and the distribution of *CCL5* haplotypes

Altogether 56% (n = 208) and 76% (n = 284) of 373 subjects were infected with HIV or HCV, respectively, and approximately half (n = 177) had HIV/HCV co-infection. More detailed description of the study population is presented elsewhere [12].

**Fig 1:**
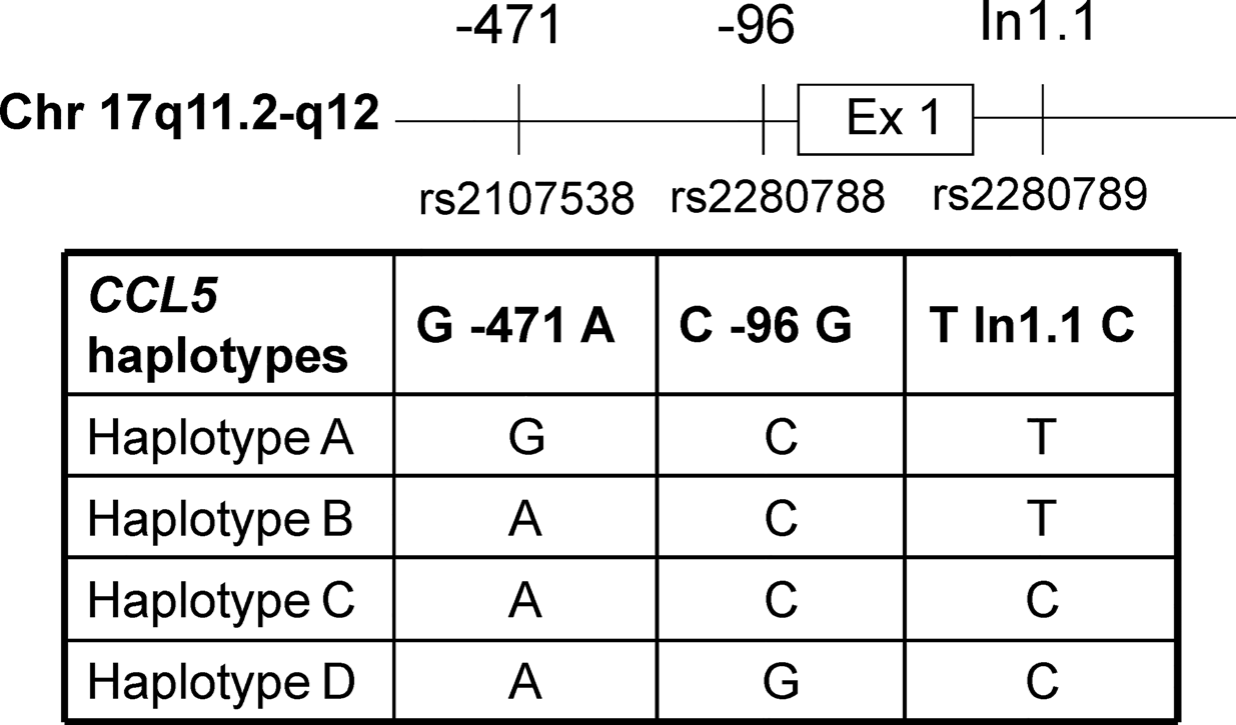
Schematic figure of *CCL5* SNPs and *CCL5* haplotypes. On the upper panel schematic figure of CCL5 gene with chromosomal location and positions of SNPs (previous codes on the top and corresponding rs-codes in the bottom) are shown. In the table letters in three right side columns indicate nucleotides at corresponding positions. Ex, exon; In, intron.

A total of 368 (98.7%) samples were successfully genotyped for *CCL5* polymorphisms and defined into the haplotypes. The overall distribution of three *CCL5* SNPs and minor allele frequencies are outlined in first row in **Table 1**. Based on these three *CCL5* polymorphisms the *CCL5* haplotypes A-D were defined (**Fig 1)**. The most common *CCL5* haplotype was haplotype A (96.2%) followed by haplotype C (14.4%), B (12.5%) and D (8.4%). Accordingly, the most frequent haplotype pair was A/A accounting for 67.4% followed by A/C (11.1%), A/B (9.8%), A/D (7.9%), B/C (2.2%), C/C (0.8%) and B/B, B/D, C/D (all 0.3%).

**Table 1:** The distribution of *CCL5* SNPs rs2107538, rs2280788, rs2280789 genotypes in people who inject drugs (PWIDs).

	rs2107538				rs2280788				rs2280789			
	MAF	GGn %	GAn %	AAn %	MAF	GGn%	GCn%	CCn%	MAF	TTn%	TCn%	CCn%
**All PWIDs**n = 368	0.18	25168.20	10628.8	143.80	0.04	33891.85	318.42	0	0.12	28577.44	7921.47	41.09
**HIV- PWIDs**n = 163	0.18	11067.48	4829.45	53.07	0.06	14689.57	1811.04	0	0.12	12476.07	3823.31	10.61
**HIV+ PWIDs**n = 205	0.19	14168.78	5828.29	94.39	0.03	19293.66	1363.41	0	0.11	16178.54	4120.00	31.46
**HCV- PWIDs**n = 87	0.18	5765.52	2933.33	11.15	**0.10**	**6979.31**	**1820.69**	**0**	0.14	6372.41	2326.44	11.15
**HCV+ PWIDs**n = 280	0.18	19067.86	7727.50	134.64	**0.03**	**26795.36**	**134.64**	**0**	0.11	22178.93	5620.00	31.07

NOTE. Significant difference (p<0.0001) between HCV seropositive and HCV seronegative PWIDs is indicated in bold.

### *CCL5* SNPs and HIV or HCV serostatus

The distribution of *CCL5* SNPs was similar between HIV seropositive and seronegative subjects. Only the prevalence of rs2280788 GC genotype was higher in HCV negative compared to HCV seropositive subjects (**Table 1**).

### *CCL5* haplotypes and HIV or HCV serostatus

**Fig 2:**
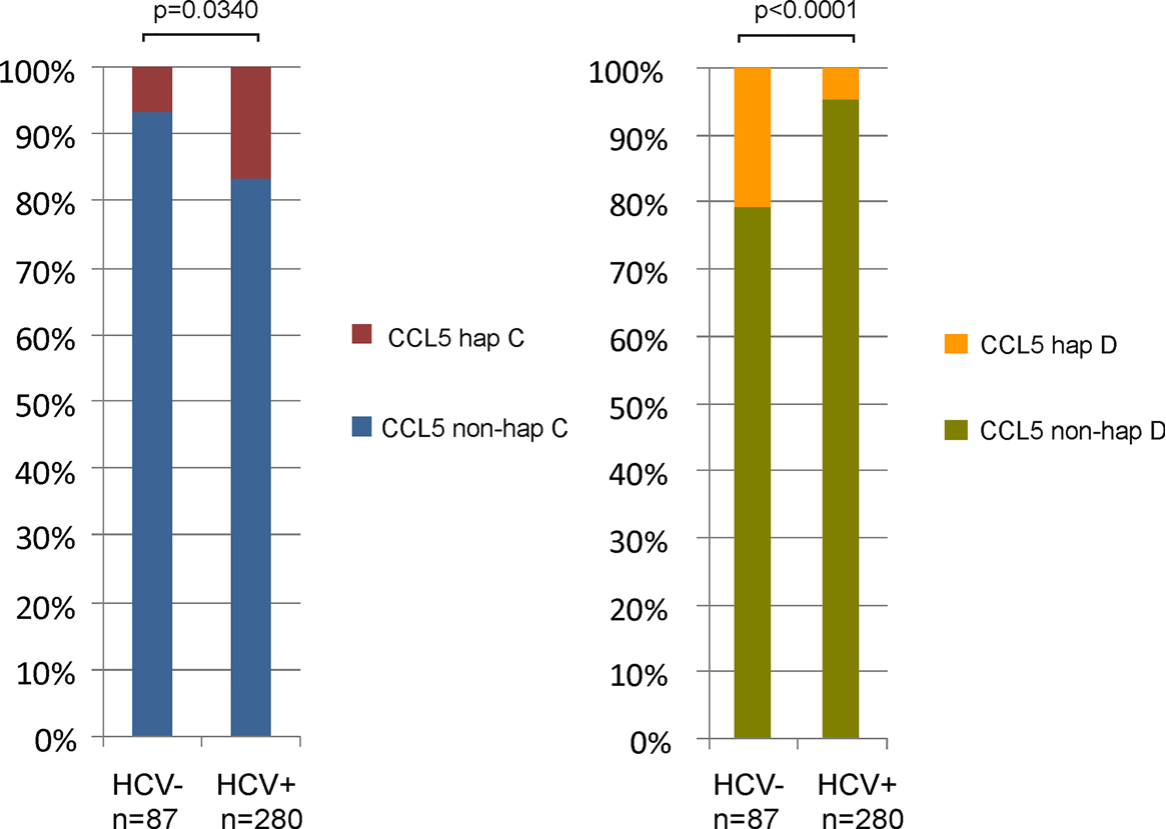
The distribution of *CCL5* haplotype C and D among HCV seronegative and seropositive PWID. hap, haplotype.

The distribution of *CCL5* haplotypes in HIV seronegative and HIV seropositive subjects was similar (data not shown). However, the frequency of *CCL5* haplotype C was lower in HCV seronegatives compared to HCV seropositives (6.9% vs. 16.8%; p<0.05) and the frequency of *CCL5* haplotype D was higher in HCV seronegatives than in the HCV seropositives (20.7% vs. 4.5%; p<0.05) (**Fig 2**). There were no differences between HCV seronegative and HCV seropositive subjects in terms of *CCL5* haplotype A and B.

### *CCL5* haplotype C and D, and HCV serostatus

Due to the different distribution previously mentioned we developed univariate and multivariate logistic regression models to evaluate how the *CCL5* haplotypes C and D influence the susceptibility to HCV infection. We observed that subjects possessing *CCL5* haplotype C had almost three times increased odds of being HCV seropositive compared to those not possessing *CCL5* haplotype C. At the same time, subjects with *CCL5* haplotype D had five times decreased odds of HCV seropositivity compared to subjects without *CCL5* haplotype D (**Table 2,** model 1). Both these associations remained significant after adjustment for covariates such as gender, age, HIV, HBV serostatus and the duration of IVDU (**Table 2,** model 2). When both haplotypes were included into the same model only haplotype D remained significantly associated with HCV serostatus suggesting the predominant influence of *CCL5* haplotype D (**Table 2**, model 3).

**Table 2:** Associations between *CCL5* haplotype C and D and HCV serostatus among people who inject drugs by univariate and multivariate models.

Models	Unadjusted	Adjusted^a^
	OR (95% CI)	OR (95% CI)
**MODEL1**		
*CCL5* hap C^b^	**2.72** (1.12–6.61)	**4.73** (1.00–22.27)
**MODEL2**		
*CCL5* hap D^c^	**0.19** (0.09–0.40)	**0.08** (0.02–0.29)
**MODEL3**		
*CCL5* hap C^b^	2.37 (0.97–5.81)	3.02 (0.63–14.59)
*CCL5* hap D^c^	**0.20** (0.09–0.43)	**0.09** (0.03–0.35)

NOTE. Significant associations between haplotypes and HCV serostatus are indicated in bold.

^a^adjusted for gender, age, HIV and HBV serostatus and the duration of intravenous drug use

^b^reference group is *CCL5* non-hap C

^c^reference group is *CCL5* non-hap D

hap, haplotype.

### *CCL5* haplotype D and HIV/HCV co-infection

**Fig 3:**
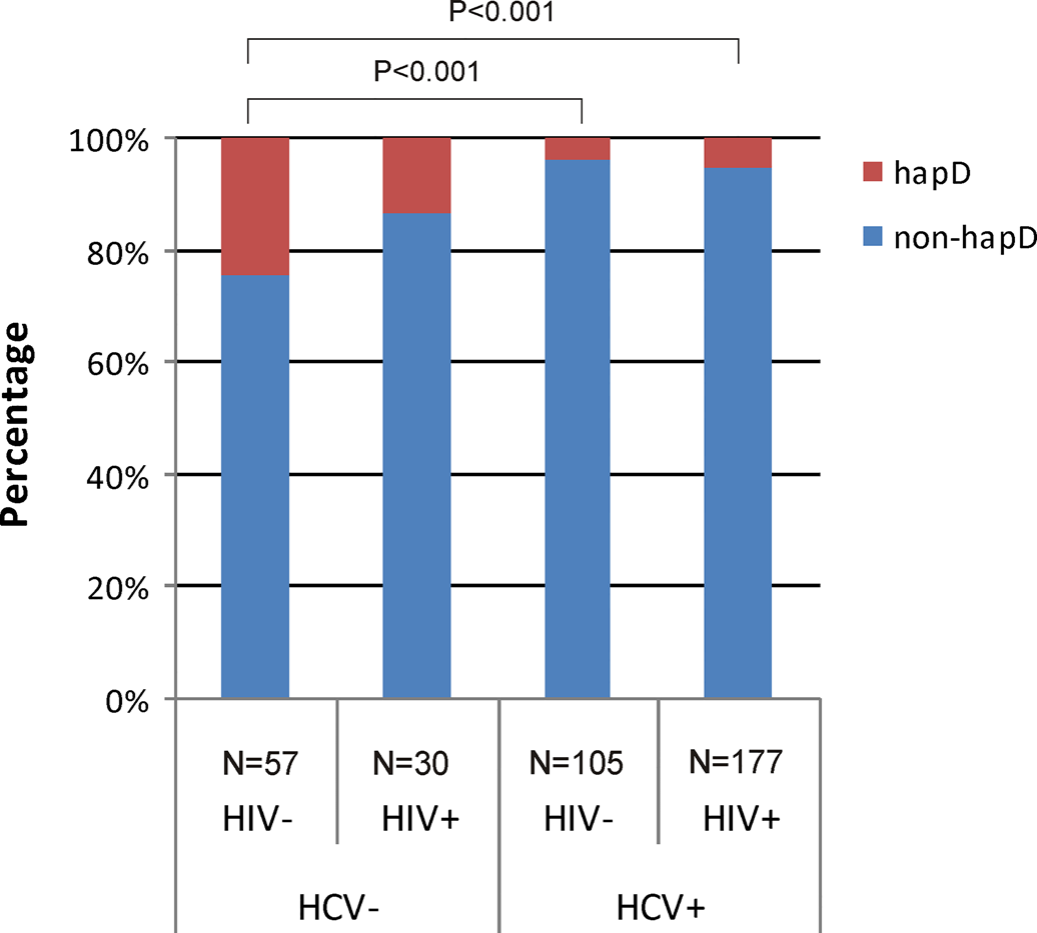
The distribution of *CCL5* haplotype D in the study population according to HIV/HCV co-infection status. hap, haplotype.

The frequency of *CCL5* haplotype D in HIV or HCV monoinfected PWID compared to HIV/HCV co-infected PWID was similar. However, *CCL5* haplotype D in HCV seronegative subjects was represented in significantly higher frequency than HCV seropositive subjects among HIV seronegative PWID but not among HIV seropositive PWID (**Fig 3**). In addition, HIV/HCV co-infected subjects possessed *CCL5* haplotype D at lower frequency than double negative subjects.´

### *CCL5* haplotype D, *CCR5* HHG*1 and HCV serostatus

The CCL5 is the potent ligand of the HIV major coreceptor CCR5 [1] and in our previous study of the same population we showed that the presence of *CCR5* HHG*1 was associated with decreased odds of HCV seropositivity [12]. For that reason we tested whether both, *CCL5* haplotype D and *CCR5* HHG*1, possess a protective effect against HCV infection. After using step-wise logistic regression analysis with backward elimination (including all co-variates), *CCL5* haplotype D, *CCR5* HHG*1 and the duration of IVDU remained significantly associated with HCV serostatus. We saw that the first two decreased the odds of HCV seropositivity and longer duration of IVDU increased the odds of being HCV seropositive (**Table 3**).

**Table 3:** Associations between *CCL5* haplotype D and *CCR5* HHG*1 on HCV serostatus in people who inject drugs by step-wise logistic regression analysis with backward elimination.

Variables	Outcome: HCV seropositivity OR (95% CI)
*CCL5* hap D^a^	0.28 (0.09–0.92)
*CCR5* HHG*1^b^	0.23 (0.08–0.68)
Duration of IVDU^c^	1.25 (1.12–1.38)

^a^reference group is *CCL5* non-hap D

^b^reference group is *CCR5* non-HHG*1

^c^continues variable, measured in years

IVDU, intravenous drug use

hap, haplotype; HH, human haplotype.

## Discussion

To the best of our knowledge, this is the first study conducted in an IDU population to evaluate the effect of *CCL5* haplotypes on the susceptibility of HCV and HIV infection. We demonstrate the involvement of *CCL5* haplotype D (rs2107538A/rs2280788G/rs2280789C) in the susceptibility of HCV such that persons possessing *CCL5* haplotype D have reduced odds of being HCV seropositive as compared to those not possessing this haplotype. Furthermore, analyzing *CCL5* haplotype D and *CCR5* HHG*1 in the same model we saw that both of them had independent protective effect against HCV. This finding makes biological sense given the potent receptor ligand nature of CCR5 and CCL5. However, in contrast to some previous studies [17, 18] we failed to find any associations between *CCL5* haplotypes and the susceptibility to HIV infection in Caucasian PWID.

We are not aware of any studies evaluating the association between *CCL5* SNPs or haplotypes and HCV susceptibility despite the fact that CCL5 participates in viral clearance during both acute and chronic HCV infection [19]. For example, patients with *CCL5* rs2280789C had an improved response to interferon therapy and those with *CCL5* rs2107538A showed milder portal inflammation than subjects without these polymorphisms [20, 21]. In fact, *CCL5* haplotype D is the combination of these two SNPs plus rs2280788G (**Fig 1**). While rs2107538A and rs2280788G have been associated with up-regulation of *CCL5* transcriptional activity, rs2280789C down-regulates this [10, 11]. However, the effect of *CCL5* haplotypes on gene expression is unknown.

We have previously demonstrated the protective effect of *CCR5* HHG*1 in the acquisition of HCV infection [12]. In the present study we show that the possession of *CCR5* HHG*1 and *CCL5* haplotype D independently protects from HCV infection. Our results suggest that despite CCR5 not being the receptor for HCV entry, the CCR5-CCL5 system is still essential in the susceptibility to HCV infection. Thus far the role of the Th1-CCL5-CCR5 system in the induction of immunity against HCV and the effect on the outcome of chronic HCV has been shown. The studies have hypothesized that CCR5 interacts with its ligands to elevate the recruitment of Th1 cells into the liver and, with this, mediates the clearance of HCV infected hepatocytes [22, 23]. The data also indicate that the lack of Th1-type cytokines favors the establishment of chronic HCV infection [24]. In addition, HCV itself down-regulates CCR5 expression via direct interaction of the HCV E2 envelope protein with tetraspanin CD81 and through HCV core protein and NS5A increase the level of CCL5 [25, 26] [27]. This leads to decreased CCR5 surface density [26] and possibly through that affects the Th1-CCL5-CCR5 system. Still, it remains unclear whether CCL5 and CCR5 directly participates in elimination of the virus or not, and what is the role of genetic variance of CCL5-CCR5 in this interaction.

We acknowledge that another host genetic factor, the polymorphism rs12979860 upstream of interleukin-28B (IL28B; also known as interferon-λ3) gene has been associated with HCV spontaneous clearance and the outcome of interferon based treatment [28–30]. One can speculate that IL28B genetic variability might also influence the susceptibility to HCV infection and therefore affect the results of the association between *CCL5* and *CCR5* haplotypes and HCV acquisition. However, we have shown that *IL28B* rs12979860 genotypes do not have an impact on HCV acquisition in Caucasian PWID population from Estonia [31]. Thus, we consider *IL28B* rs12979860 unlikely to interact with the results of current study.

Based on the previously described associations between *CCL5* SNPs and susceptibility to HIV infection, we expected to observe differences in the distribution of *CCL5* haplotypes between HIV negative and HIV positive subjects [10, 17, 18]. In contrast, our data seems to confirm the findings of Liu *et al* (1999) in haemophilia patients, which indicate that there is no association between *CCL5* polymorphisms and susceptibility to HIV [11]. On the one hand, the potential effect may be shaded by the extent of viral exposure. Intravenously infected patients are exposed to a much greater amount of viruses than those infected through other routes [32]. On the other hand, the population studied in here is extremely hard to recruit and we acknowledge that associations in this group might be insignificant due to low power. Therefore, we cannot draw final conclusions in the context of HIV infection.

Some limitations of the study should be acknowledged. First, the duration of IVDU was only known for two thirds of the population (lacking from prison population). However, we assume that the duration of IVDU in the syringe exchange population reflects the IVDU in the prison population because these persons are similar in terms of demographic and risk behaviors as well as the short duration of the HIV epidemic in Estonia in general (Zettenberg et al., 2004, Adojaan et al., 2005). Second, the lack of information on disease stages of HIV and HCV infection reflects the seroprevalent nature of the cohort and thus we were not able to analyze the effect of haplotypes on disease progression. In addition, previous studies have shown the association of HCV clearance with genetic diversity of hepatitis A virus cellular receptor 1 and HCV genotypes [33]. Unfortunately, there was no data available with regard to HCV RNA and HCV genotypes, so we could not analyze the associations between HCV clearance, HCV genotypes and *CCL5* haplotypes. However, from the demographic background we know that in Estonia, the main HCV genotype is Ib (60% to 70%) followed by IIIa (around 30%) in HCV monoinfected as well as in HIV/HCV co-infected population [34, 35]. Third, we were unable to draw conclusions with regard to the association between *CCL5* haplotype D and HIV/HCV co-infection because of low proportion of HIV monoinfected subjects. Lastly, we acknowledge that we do not have access to *CCL5* haplotype frequency in well-controlled normal population. However, 24% of our studied population is HCV seronegative and 16% of them are seronegative for both HCV and HIV. They had same demographic and similar risk behavior characteristics compared to the HCV seropositive population. In addition, we found that the *CCL5* haplotype frequency in our studied population is comparable to other presumably HIV and HCV Caucasian populations (www.hapmap.org). Thus, the likelihood of our findings to be confounded due to the lack of control population is very low. Nevertheless, we believe that these limitations do not diminish the importance of our results.

In conclusion, we suggest that in PWID exposed to HIV and HCV, *CCL5* haplotype D (-471A/-96G/In1.1C) and *CCR5* HHG*1 protect against HCV acquisition. Our findings highlight the importance of *CCL5* variability and the CCL5-CCR5 axis on HCV acquisition.

## Supporting Information

S1 Table Studies of associations between polymorphisms in *CCL5* and acquisition of HIV-1 or HCV disease progression. (DOC)
