# Supplementary material for: A CCL5 Haplotype Is Associated with Low Seropositivity Rate of HCV Infection in People Who Inject Drugs
Source: PLoS One. 2016 Jun 15;11(6):e0156850. doi: 10.1371/journal.pone.0156850 (PMC4909289; doi:10.1371/journal.pone.0156850)
Supplement: S1 Table — (DOC) [file pone.0156850.s001.doc]

**Supplementary Table 1. Studies of associations between polymorphisms in *CCL5* and acquisition of HIV-1 or HCV disease progression**

| **HIV INFECTION** | | | | |
| --- | --- | --- | --- | --- |
|  | ***CCL5* SNP (rs-code) /haplotypes** | **Effect on susceptibility** | **Study population; risk group** | **Reference** |
| ***CCL5***  **SNP** | **TIn1.1C (rs2280789)** | | | |
|  | T | Higher risk | Indians;  sexual | [1] |
|  | C | Higher risk | AA, EA, Asians  Mixed group* | [2] |
|  | **G-471A (rs2107538)** | | | |
|  | **A** | Higher risk | AA, EA, Asians, Caucasians;  Mixed, Sexual (Mcdermott),  Sexual/heamophiliacs (Ahlestiel) | [2-4] |
|  | **T3’-222C (NA)** | | | |
|  | C | Higher risk | AA, EA, Asians;  Mixed group* | [2] |
| ***CCL5* haplotype** | **G-471A/C-96G (rs2107538/rs2280788)** | | | |
|  | A/C | Higher risk | EA;  ND | [5] |
|  | GG/CC | Lower risk | EA;  sexual | [4] |
|  | **G-471A/C-96G/TIn1.1C (rs2107538/rs2280788/rs2280789)** | | | |
|  | A/C/T | Higher risk | Indians;  sexual | [1] |
| **HCV INFECTION** | | | | |
|  | **CCL5 SNP/haplotype** | **Effect on disease progression** | **Study population**** | **Reference** |
| ***CCL5***  **SNP** | **G-471A (rs2107538)** | | | |
|  | A | Milder portal inflammation | Caucasians | [6] |
|  |  | Less severe hepatic inflammation | Caucasians | [7] |
|  | **TIn1.1C (rs2280789)** | | | |
|  | C | Worse response to interferon therapy | Caucasians | [8] |
|  | **T3’-222C (NA)** | | | |
|  | C | Worse response to interferon therapy | Caucasians | [8] |
| ***CCL5* haplotype** | **G-471A/TIn1.1C/T3’222C (rs2107538/rs2280789/ NA)** | | | |
|  | Combination of A/C/T and A/C/C | Worse response to interferon therapy | Caucasians | [8] |

NOTE.

*study population consisted different risk groups and they all were analysed together

** the risk group is not defined in these studies

AA, African-Americans; EA, European-Americans; SNP, single nucleotide polymorphism; ND, not defined; NA, not available

References:

1. Rathore A, Chatterjee A, Sivarama P, Yamamoto N, Singhal PK and Dhole TN. Association of RANTES -403 G/A, -28 C/G and In1.1 T/C polymorphism with HIV-1 transmission and progression among North Indians. J Med Virol 2008;80:1133-41

2. An P, Nelson GW, Wang L, et al. Modulating influence on HIV/AIDS by interacting RANTES gene variants. Proc Natl Acad Sci U S A 2002;99:10002-7

3. Ahlenstiel G, Iwan A, Nattermann J, et al. Distribution and effects of polymorphic RANTES gene alleles in HIV/HCV coinfection -- a prospective cross-sectional study. World J Gastroenterol 2005;11:7631-8

4. McDermott DH, Beecroft MJ, Kleeberger CA, et al. Chemokine RANTES promoter polymorphism affects risk of both HIV infection and disease progression in the Multicenter AIDS Cohort Study. Aids 2000;14:2671-8

5. Gonzalez E, Dhanda R, Bamshad M, et al. Global survey of genetic variation in CCR5, RANTES, and MIP-1alpha: impact on the epidemiology of the HIV-1 pandemic. Proc Natl Acad Sci U S A 2001;98:5199-204

6. Hellier S, Frodsham AJ, Hennig BJ, et al. Association of genetic variants of the chemokine receptor CCR5 and its ligands, RANTES and MCP-2, with outcome of HCV infection. Hepatology 2003;38:1468-76

7. Promrat K, McDermott DH, Gonzalez CM, et al. Associations of chemokine system polymorphisms with clinical outcomes and treatment responses of chronic hepatitis C. Gastroenterology 2003;124:352-60

8. Wasmuth HE, Werth A, Mueller T, et al. Haplotype-tagging RANTES gene variants influence response to antiviral therapy in chronic hepatitis C. Hepatology 2004;40:327-34
